# A values-driven academic affiliation between a public medical school and a private healthcare provider: exploring the perceptions of key opinion leaders

**DOI:** 10.3389/frhs.2025.1655759

**Published:** 2025-08-28

**Authors:** Leon Du Preez, Farah Otaki, Timo Clemens, Suleiman Al-Hammadi, Adrian Stanley, Samuel B. Ho, Paddy Kilian, Pietie Loubser, Riad Bayoumi, Mutairu Ezimokhai, Barry Bedford, Tarek Fathey, Reem AlGurg, Hanan Al Suwaidi, Amer A. Sharif, Alawi A. Alsheikh-Ali

**Affiliations:** ^1^ Provost Office, Mohammed Bin Rashid University of Medicine and Health Sciences, Dubai Health, Dubai, United Arab Emirates; ^2^ Mediclinic Middle East, Dubai, United Arab Emirates; ^3^ Strategy and Institutional Excellence, Mohammed Bin Rashid University of Medicine and Health Sciences, Dubai Health, Dubai, United Arab Emirates; ^4^ Department of Health Services Research, Care and Public Health Research Institute (CAPHRI), Faculty of Health, Medicine, and Life Sciences (FHML), Maastricht University, Maastricht, Netherlands; ^5^ Department of International Health, Care and Public Health Research Institute (CAPHRI), Faculty of Health, Medicine, and Life Sciences (FHML), Maastricht University, Maastricht, Netherlands; ^6^ College of Medicine, Mohammed Bin Rashid University of Medicine and Health Sciences, Dubai Health, Dubai, United Arab Emirates; ^7^ President Office, Mohammed Bin Rashid University of Medicine and Health Sciences, Dubai Health, Dubai, United Arab Emirates; ^8^ Dubai Health, Dubai, United Arab Emirates

**Keywords:** value-based health care, Academic Health System, public private affiliation, Sustainable Development Goals, SDG 3, SDG 4, and SDG 9, and SDG 17, Dubai Health, United Arab Emirates, public health

## Abstract

**Introduction:**

In an Academic Health System model where university and clinical care institutions are separate entities, robust agreements are needed for effective working relationships among the involved institutions. There is paucity in the literature around reports of such affiliations, especially those relating to public private partnerships. Accordingly, the overall purpose of this study is to explore the perception of key opinion leaders about the development of a values-driven affiliation between a public medical school and a private healthcare provider in the first integrated Academic Health System in Dubai, United Arab Emirates, namely: Dubai Health. The process of developing the respective affiliation is based on the principles of action research. It involved ongoing cycles of planning, acting, observing, and reflecting.

**Methods:**

This study relied on a qualitative phenomenological research design, where 18 primary stakeholders, who played an active role in making the affiliation, were given the option of providing their feedback either in writing, using a tailor-made questionnaire, or in the form of a semi-structured interview. Constructivist epistemology constituted the basis of the entailed interpretive qualitative analysis, which followed the six-step analysis approach initially introduced by Braun and Clarke (2006).

**Results:**

The qualitative analysis led, as per this study's conceptual framework: “Public Private Affiliation Journey”, to two interconnected themes, namely: Key Milestones and Driving Forces. Within Key Milestones, seven sequential categories were identified: Observing a triggering need, Finding a good match, Seizing the opportunity, Arriving at a common ground, Looking ahead, Venturing for the right reasons, and Reaping the benefits. Within the second theme: Driving Forces, the following three categories were identified: Aspiring for success, Leveraging human qualities, and Doing things the right way.

**Discussion:**

This study showed that there is a latent potential in forming public private partnerships that can enable the formation and development of Academic Health Systems. It also showed how the guidelines of action research can be set as the basis of the process of partnership formation, and how following those guidelines in such an endeavor maximizes value for all. In addition, it clearly brought forth the importance of having a robust governance structure with committed and engaged leadership, and clear communication channels, and of equipping the physicians with the skills needed to be effective educators. Lastly, this study introduced the “Public Private Affiliation Journey” conceptual framework, which can be deployed in “federated” Academic Health Systems worldwide to increase the chances of success of public private partnerships and to maximize the value attained through them.

## Introduction

The Institute for Healthcare Improvement (IHI) triple aim conceptualizes optimizing the performance of any health system through the simultaneous pursuit of three dimensions: improving the patient experience of care (including quality and satisfaction), improving the health of populations, and reducing the per capita cost of health care (1). Relevantly, value-based health care is rooted in the belief that value for patients must be the ultimate goal in the organization and management of healthcare delivery systems (2, 3). Along those lines, any Academic Health System (AHS) exists for the principal objective of improving health and reducing the burden of illness in society (4). This is accomplished by a tripartite mission of (i) providing patient care, (ii) educating and training (future health) professionals, and (iii) conducting research, and translating discoveries into improved approaches to health and disease. As such, these three missions are best implemented synergistically to advance the unified purpose of attaining a healthier future for all (4). The AHS model that supports this sophisticated enterprise relies on a high degree of integration between the three mission areas (i.e., patient care, health professions education, and research), and the balanced allocation of resources. The AHS model has been commonly deployed for over at least five decades and has proven to add value (4).

AHSs are needing to adapt to various powerful global healthcare trends (5, 6). These include but are not limited to: health and care workforce shortage (7, 8); changing needs of patients; shifting payment models emphasizing efficiency and value (9, 10); continuous threat on financing streams (especially those that are meant to support health professions education and research) along with the need for intense investment in information management systems which is further straining existing financial resources (11); increasing competition; and the unprecedented pace of medical innovation.

The recognition of these issues has led to the widespread opinion that AHSs, which have been characterized as “islands of excellence” (4), need a fundamental overhaul to achieve IHI triple aim of better patient experience, higher quality care, and lower cost (1). This will require system redesign (12): reengineering perhaps all aspects of their sophisticated operations to adapt to a rapidly changing external environment that is constraining the financial resources needed to maintain and advance the three interdependent mission components (4). The university/ medical school within any AHS is in the knowledge business with value placed upon the concept of academic freedom/ independence (13). Concurrently within AHSs, the clinical care providing entities are in the highly competitive healthcare business (14). The corresponding AHS model is supposed to rely on success of the latter to support the academic and research programs conducted in the former. The cultures that characterize these two endeavors often seem like parallel universes (4). Hence, the emphasis becomes on merging the best of both, which is a prerequisite to aligning all aspects of the AHS tripartite mission.

These learned lessons and conclusions are worth leveraging in the Middle East and North Africa region (MENA) where the very concept of and experience with AHSs are still immature (15). This requires acknowledging the abovementioned challenges and seeking to solve them, and developing and maintaining good relationships between all relevant stakeholders (16). In aggregate, these developments are expected to facilitate establishing the AHSs of the future (14). Within this context, which constitutes both a challenge and an opportunity for healthcare leaders, exploring and realizing the latent potential in building system-wide partnerships become of utmost importance (17). In fact, the leadership and governance of AHSs tend to be committed to fulfilling the tripartite mission through an effective academic partnership between a university and health care related division(s) (4, 18). In what tends to be referred to as a “consolidated” AHS model, the university owns the academic hospitals, or the academic hospital operates the medical school. Both ways, the singularity of leadership and corporate ownership simplifies authority and responsibility. However, in a more “federated” AHS model, where university and clinical care institutions are separate entities, robust affiliation agreements are needed among the involved institutions to set a framework for shared academic authority and fiduciary oversight. This calls for a comprehensive affiliation agreement which extensively covers all relevant academic and clinical activities. This agreement needs to also include terms for conflict resolution in case an issue that cannot be resolved following policy and procedures arises.

Successes of such affiliations, within the context of AHSs, have been reported (19). The mindset shifts from the individual institution competitiveness to the synergy and catalytic effect that multiple institutions can achieve together to improve their community's health and economic gain (17). To the best of the authors' knowledge, there is paucity in the literature around such affiliations, especially those relating to public private partnerships. Developing a thorough understanding of such affiliations will require capturing and systematically analyzing the perspective of key stakeholders. Accordingly, the overall purpose of the study is to explore the perception of Key Opinion Leaders (KOLs) about the development of an innovative, values-driven affiliation (that aimed primarily at enabling the clinical placements integral to an undergraduate medical program) between a public medical school and a private healthcare provider in an AHS in Dubai.

## Methods

### Context of the study

United Arab Emirates (UAE) is a multi-cultural, multi-ethnic setting in the MENA, rooted in Islamic values and Arabian traditions. UAE is a constitutional federation of seven emirates. Abu Dhabi city is the capital of the UAE federation (20), and Dubai is the largest and most populous of the seven emirates (21). According to the most recent Annual UAE Economy Report, GDP at constant prices amounted to AED 1,418.9 billion for the year 2020, while GDP at current prices amounted to AED 1,317.9 billion (22). The UAE territory is approximately 71,023.6 sq km of land, including some islands in the Arab Gulf, in addition to 27,624.9 sq km of territorial water. All UAE citizens in the seven emirates carry the unified nationality of the UAE, which is recognized internationally (20). According to the Federal Competitiveness and Statistics Centre, the UAE's total population (nationals and expatriate residents) was 9,282,410 in 2020 (23). The UAE is considered one of the most unified and trusted countries in the world, according to the 2023 Edelman Trust Barometer (24). The respective global index measures trust across four institutions: government, business, Non-Governmental Organizations (NGOs), and media. The government once more topped the list as the most trusted institution. UAE residents, in general, and those in Dubai, in specific, have strong trust in the government.

Named after His Highness Sheikh Mohammed Bin Rashid Al Maktoum, Vice President and Prime Minister of the UAE, and Ruler of Dubai, Mohamed Bin Rashid University of Medicine and Health Sciences (MBRU) was set-up in September 2014 (25). In November 2014, provisional approval of MBRU as a Higher Education Institution (HEI) was granted by the Commission for Academic Accreditation (CAA): UAE's federal government quality assurance agency for higher education. In December 2014, the license was received from the UAE Ministry of Higher Education and Scientific Research. Queen's University Belfast (QUB) partnered with MBRU as principal curriculum advisers in January 2015. MBRU was officially established by royal decree in June 2016.

**Figure 1:**
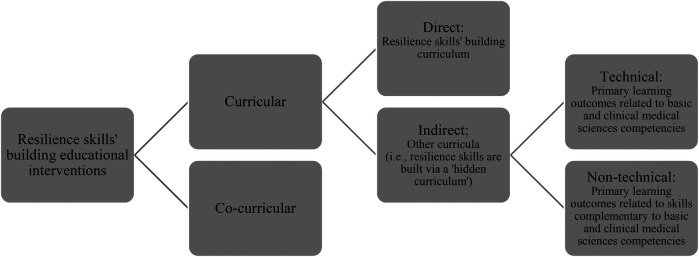
Outline of learning and teaching interventions at MBRU, that are continuously developing through design-based research, aimed at building resilience skills through fostering self-regulated learning.

MBRU currently has three colleges: College of Medicine (CoM), Hamdan Bin Mohammed College of Dental Medicine (HBMCDM), and Hind Bint Maktoum College of Nursing and Midwifery (HBMCoNM). MBRU's culture is characterized by evidence-based decision-making and effective deployment of ongoing cycles of implementation science, including but not limited to action research and design-based research (26–33). The affiliation that a segment of which is investigated in the current study is an example of a collective experience that has been following the principles of action research. As for design-based research, it is the means by which MBRU continuously improves the practice (while contributing to theory of the subject matter) around innovative (commonly homegrown and “greenfield”) interventions, learning and teaching or otherwise (34–37). MBRU's commitment to continuously developing and to building resilience is also evident through its flagship learning and teaching interventions that are implemented (in alignment with the principles of design-based research, as well) to, either directly or indirectly, fostering self-regulated learning and building resilience among learners (Figure 1). These interventions include a homegrown curricular course that is offered to learners to inspire and empower them to build their own resilience skills (28, 38). There are other interventions offered by MBRU that foster individual and collective resilience indirectly through curricular [e.g., professionalism training (26)] and co-curricular self-regulated learning. An example of a flagship co-curricular program would be the MBRU- Summer Scholars' Program that supports the learners in their journey towards becoming global citizens (30, 31).

The Bachelor of Medicine, Bachelor of Surgery degree (MBBS), offered at MBRU, consists of a six-year curriculum, built on a competency-based learning model. The MBBS is comprised of three phases. The learning process is spiral (39), with courses integrated across six academic years, each of two semesters. The first year constitutes Phase 1 and exposes the students to basic concepts of medicine. Phase 2 covers the second and third years, where teaching is organized around body organ systems, integrated with clinical medicine. The fourth through sixth years represent Phase 3 during which the students undergo their clinical rotations. The first cohort of 56 medical students were enrolled in August 2016.

MBRU had set out to become an integrated AHS, with clear articulation of its direction in the University's goal upon inception:

‘To advance health in the UAE and the region, through an innovative and *integrated academic health system*, that is nationally responsive and globally connected, serving individuals and communities.’

**Figure 2:**
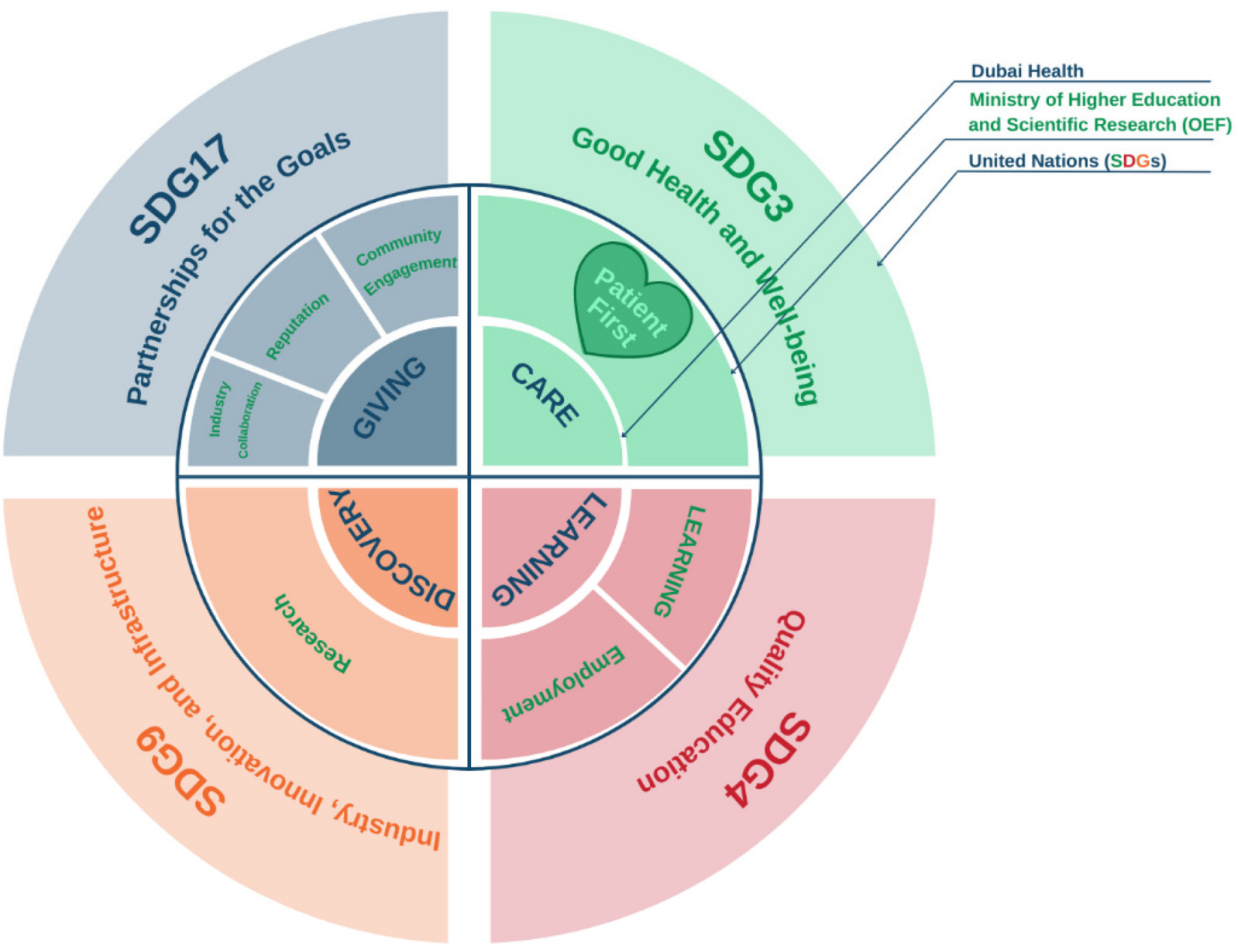
Alignment of goals (Micro-, Meso-, and Macro-levels), first shared via a report on a mixed methods study investigating an innovative, continuously developing learning and teaching intervention (following design-based research) which is implemented in MBRU (33). The figure shows how the pillars of Dubai Health (Care, Learning, and Discovery, and Giving) are feeding into the pillars of the Outcome-based Evaluation Framework (OEF) which in turn contribute to select Sustainable Development Goals (SDGs). Patient first, as the core value of Dubai Health [purposely located in the heart of the illustration, slightly towards the left (in Green, which is considered a balanced anchor for the other colors of the visible spectrum)]. This patient centricity is among the differentiators of MBRU from the rest of the Higher Education Institutions governed by the OEF of the UAE Ministry of Higher Education and Scientific Research.

Today, MBRU is the implementation vehicle for the “learning” (i.e., health and care professions' education) and “discovery” (i.e., research) arms of the first integrated academic health systems in Dubai, namely: Dubai Health (40). Besides those two missions, Dubai Health oversees the operations of around 40% of the health sector in Dubai through its clinical enterprise (i.e., the “care” arm). The rest of the health sector in Dubai is mainly privately owned and operated. In its entirety, Dubai's healthcare sector is regulated by the Dubai Health Authority (DHA). Besides, the “care”, “learning”, and “discovery” arms, that can be mapped onto the traditional tripartite mission of the common academic health centers in North America (4), Dubai Health also has an arm related to philanthropy namely: “giving”. Dubai Health is characterized by a values-driven culture. The institution's core value is “Patient First”. The other values are “Respect”, “Excellence”, “Teamwork”, “Integrity”, and “Empathy”. In addition to sharing its goals with Dubai Health, MBRU efforts align nationally with the UAE Ministry of Higher Education and Scientific Research- Outcome-based Evaluation Framework (41) and internationally with the United Nations- Sustainable Development Goals (SDGs) (Figure 2) (42).

Mediclinic International (MCI), subsequently renamed Mediclinic Group (MCG) (43), is an international healthcare provider (established in 1983) which owns 74 hospitals with divisions in South Africa, Switzerland, and Middle East. The Founder of MCI has embraced and supported the vision of training medical students since the establishment of MCI. In the UAE, Mediclinic Middle East (MCME) has 7 hospitals, 3 of which are in Dubai (Mediclinic City, Mediclinic Parkview, and Mediclinic Welcare Hospitals), and 29 ambulatory care clinics of which 16 are in Dubai including one large ambulatory center: Mediclinic Dubai Mall (44, 45). MCME is considered among the largest healthcare delivery networks in the UAE, and has a capacity of around 1,000 inpatient beds and 1,300 doctors (21, 45). The network offers a full range of preventive and curative services including primary, secondary, tertiary, and quaternary.

At the time of the affiliation reported upon in the current manuscript, DHA and the Dubai Healthcare City Authority (DHCA) constituted mutually exclusive regulators. Mediclinic City Hospital was governed by DHCA [given its location in Dubai Healthcare City (DHCC)] and the rest of the units by DHA (located outside DHCC). There has been a growing trend of public private partnerships in Dubai across all industries, and the affiliation (reported upon in the current manuscript) is a leading example in the education and/ or health sectors.

### Developing the MBRU-MCME affiliation

The process of developing the MBRU-MCME affiliation has been based on the principles of action research (46–48). It involves investigations, reflections, and improvements of practice, through ongoing cycles of planning, acting, observing, and reflecting, as described below [following Template for Intervention Description and Replication (TIDieR) guidelines (49, 50)].

### Planning

Given the established need for MBRU to have a site for clinical placement for students in the MBBS, MBRU leadership approached MCME leadership to initiate a discussion around the potentiality of developing a mutually beneficial collaboration. This happened soon after launching the MBBS, and hence time was a critical factor (i.e., enrolled students needed clinical exposure early in their program and full clinical clerkships by the fourth year).

As such, a team with representation from both entities (i.e., MBRU and MCME), led by the Founding Dean of CoM at MBRU and Senior Corporate Medical Director of MCME, was assembled to formalize and strategically oversee the initial affiliation agreement. Other members of the team included the Provost and Chairperson of Basic Sciences from MBRU, and Chief Operating Officer and Chief Clinical Officer from MCME.

The collaboration was initiated by signing a Memorandum of Understanding in April 2015, followed by the development and endorsement of an initial, fixed-term affiliation agreement of three years in November 2016. At that time, MBRU was composed of two colleges: CoM and HBMCDM.

The purpose of this agreement was to address MBRU's pressing need for a clinical training site, along with enabling MCME units to become academic providers of care. This included providing the optimum environment (within preset guidelines) for conducting experiential learning for future doctors. The focus of the agreement was the placement of students for their clinical clerkship in years 4, 5, and 6 as an integral part of the MBBS. One of the byproducts of the developed relationship between both entities was the opportunity for interested MBRU in-house faculty to have a clinical practice in MCME.

Under the agreement, MBRU's responsibilities included:

- Administering and assuring the quality of the MBBS
- Providing the curricular requirements for the clerkships
- Ensuring the attainment of prerequisite competencies (among the learners) prior to entering the clinical placements
- Providing the criteria for students' assessment and progression
- Appointing MCME physicians as adjunct faculty to train learners
- Providing structured faculty development for adjunct faculty
- Providing medical liability insurance for the learners

As for MCME's responsibilities, they included:

- Developing the clerkship curricula, in collaboration with MBRU
- Integrating students in the facility operations, including but not necessarily limited to: orientation and onboarding (similar to that of MCME employees), identification card, utilization of the Electronic Health Records, and participation in quality, patient safety, infection control initiatives, and multidisciplinary team activities
- Providing access, support services, and equipment for learners
- Offering the required experiential learning
- Assessing and reporting back to MBRU on the students' performance and progress
- Supervising the learners in the clinical setting

In terms of assuring the quality of the affiliation, the Clinical Academic Committee (CAC) with a joint representation from both MCME and MBRU was established with the mandate of providing operational oversight including monitoring and evaluating to continuously improve the student clinical training. The first meeting took place in June 2017. Soon afterwards, the need to address day-to-day issues became evident; as such, the Clerkship Management Committee (CMC) was developed to handle this ongoing responsibility through monthly meetings. The first meeting of the respective committee took place in August 2018. CMC's mechanism of operation became closely intertwined with a structure that assigned equivalent roles and responsibilities around developing and delivering the curriculum to MCME and MBRU.

With guidance from the committees, preparation for the clerkship began with joint curriculum planning, faculty development workshops, and the establishment of an organizational structure of academic, administrative, and discipline coordinators in MCME to mirror MBRU's learning and teaching structures.

### Acting

The Memorandum of Understanding was signed in April 2015 by the Managing Director of the Education Division of DHCA (who subsequently became the Vice Chancellor of MBRU) and the Chief Executive Officer (CEO) of MCME.

Soon afterwards, the initial affiliation between both entities was formalized. The affiliation agreement was signed off in November 2016. Initially, the affiliation included only Mediclinic City Hospital as a clinical training site, and subsequently other Mediclinic hospitals and clinics in Dubai were added.

MBRU effectively integrated the access to clinical sites into its educational delivery, which was marked by informing the CAA of the affiliation agreement. This was first reflected in the Academic Catalog of 2018–2019.

In preparation for the clinical rotations, MBRU in-house faculty visited all the training sites to systematically orient and build rapport with MCME adjunct faculty. This included onsite training for physician supervision and workplace-based assessment of students. To further support the MCME adjunct faculty, MBRU introduced an annual Medical Education Symposium along with the Advanced Medical Education (ACE) certification program to enable continuous faculty development. Moreover, a policy was set in place to outline the process of obtaining, at all levels and in all settings within MCME, patients' consent to participate in student clinical training. The operationalization of the policy was set to involve patients' verbal consent in the clinic setting and written consent in the inpatient setting, both of which were solicited by the nurses as a neutral party.

Later, in December 2022, the following clause was added to the General Patient Consent form:

‘I understand that medical students and trainees in health specialties might participate in my care processes, if applicable’.

In August 2019 (i.e., start of academic year 2019–2020), the first cohort of 47 year 4 students (Class of 2022) commenced their clerkship in surgery, pediatrics, internal medicine, and family medicine at MCME (and psychiatry at a specialist federal hospital). This was marked with a formal public ceremony whereby MCME senior leadership welcomed the inaugural cohort and handed over to them a badge with logos of both institutions.

MCME integrated the students into all the activities of the clinical setting which included read-only access to the Electronic Health Records, a student module (as part of the Electronic Health Records) for clinical notetaking, attendance at multidisciplinary meetings, and interaction with all facility staff. The students were also required to sign a confidentiality agreement during the onboarding process at MCME.

In March 2020, due to the onset of COVID-19, CoM at MBRU transitioned all educational activities to the online environment. The transition was based on the principles of action research, as well (37).

The impact was most significant on the phase 3 curriculum since students could not complete the last two clinical rotations. At the onset of the pandemic, year 4 students (i.e., the only cohort in phase 3) were midway through their fourth of a total of five clinical rotations. Intensive efforts were made to compensate for the lost clinical experience through innovative learning and teaching modalities (51). A 3-week “enhanced induction” was introduced at the beginning of the following academic year, which was designed to address the identified deficiencies in clinical experiential learning for year 4 students, and thus focused on their missed rotations. The continuous adjunct faculty development (i.e., ACE program) was maintained through the online environment.

When the first, abovementioned cohort progressed to year 5 (i.e., academic year 2020–2021), MCME accommodated (while enforcing all the necessary COVID-19 precautions) emergency medicine, otorhinolaryngology, intensive care, neurology, obstetrics and gynecology, orthopedics, urology, and vascular surgery clinical rotations. Some of the year 5 clinical placements took place in the public hospitals (a portion of the emergency medicine, neurology, obstetrics and gynecology, and ophthalmology, and all of the pediatrics rotations).

In year 6 (i.e., academic year 2021–2022), those students entered a structured apprenticeship in family medicine, internal medicine, surgery, pediatrics, and obstetrics and gynecology which includes further learning and an increased level of patient care at MCME. There was an equal distribution of students in MCME and the public sector hospitals, with MCME accepting students in all but the family medicine specialty.

### Observing and reflecting

The MBRU-MCME-affiliation-steering-team maintained oversight by regularly convening to reflect upon performance and progress, and to collectively attend to arising challenges.

Initially, it was envisaged that MBRU would be reimbursing MCME for the time adjunct faculty spend on teaching students. Later, the Senior Corporate Medical Director recommended not to endorse the financial element given the difficulty around finding a formula that accurately quantifies teaching time (for reimbursement purposes), which also coincided with the shift in focus, wherein the potential strategic value of the affiliation overrode the transactional nature of the financial element. Concurrently, teaching workload became a formal requirement in the job descriptions of the MCME physicians. From 2018 onwards it became firmly established as a clause in physician contracts:

‘Training of Medical Students: MCME’s commitment to actively participate in Physician training in the UAE is embedded in Memorandums of Understandings with selected, accredited learning institutions. Training is conducted in a structured outcome-based manner by facilitating learning in the workplace in a responsible manner. As such, physicians are expected, as part of their normal employment with the company, to train medical students in accordance with the agreed curriculum of said tertiary education institutions.’

In general, this addition of the teaching responsibility was appreciated by healthcare providers, in turn leading to greater professional satisfaction.

The affiliation agreement was approaching expiration at the onset of COVID-19. A dialogue, around developing a new affiliation agreement, which had just started needed to pause due to the shift in priorities. Meanwhile, given the good faith between the parties, the affiliation was maintained, continuing to fulfill the clinical placement needs of the MBBS for academic years 2019–2020, 2020–2021, and 2021–2022.

By the end of the academic year 2021–2022, 40 students successfully graduated, upon completing the preset clerkship requirements (i.e., the first graduating cohort). In addition, 48 s cohort students progressed from year 4 to year 5, and 33 third cohort students progressed from year 5 to year 6. In the respective year, a total of 249 MCME adjunct faculty contributed to the teaching.

It was observed that in many instances, the affiliation with MCME strengthened components of the MBBS. For example, in a 5-course research module offered in the first through fifth semesters of the MBBS (39), a total of 65 student research projects were supervised by MCME adjunct faculty. Relevantly, the annual MCME research conference constituted an opportunity for students to present their research work. The affiliation also created additional local co-curricular placement opportunities within MCME as part of one of the University's flagship program, namely: MBRU-Summer Scholars' Program (29, 31, 52), which also included placements in MCME hospitals in Abu Dhabi. Additionally, the affiliation contributed to broadening the horizons of the students since it gave them insight into the practice of medicine in the private sector.

### Research design

This research study relied on a phenomenological research design, which has been suggested to constitute a pragmatic approach (along with grounded theory and framework analysis) to analyzing qualitative data around implementation sciences (53, 54). Qualitative methods are increasingly used in relation to implementation sciences primarily because they enable understanding sophisticated systems involving diverse stakeholders (e.g., *value-driven learning academic health systems*) (55, 56). Many researchers are new to these methods (57–59) and not necessarily aware of the flexibility afforded (and worth leveraging) by applied qualitative research (55, 60). According, implementation scientists are encouraged to benefit from guidance on creating a pragmatic approach to analysis, which includes the strategic combining and borrowing from established approaches to meet a given study's needs, typically with guidance from an implementation science framework (e.g., action research and design-based research), and explicit practice and research change goals (e.g., enabling the clinical placements integral to a medical program, and contributing to the literature in relation to how “federated” AHSs worldwide can increase the chances of success of public private partnerships) (61–63). From this perspective, the study followed established guidelines of how to use pragmatic analytic approaches to meet the needs and constraints of implementation science initiatives while maintaining and enabling communication of the entailed research work's rigor (53). Constructivist epistemology constituted the basis of the entailed interpretive qualitative analysis (64), which followed the six-step analysis approach initially introduced by Braun and Clarke (2006) (65–67). This multi-phased methodology to inductive qualitative analysis is encouraged in socio-behavioral research (68). By leveraging this participant-focused research design (69), the researchers were able to tap into the participants' lived experiences of developing a values-driven affiliation between a public medical school and a private healthcare provider. The participants had complete autonomy to choose whether, or not, to participate in the study. The study was approved by three relevant research governing entities: the Institutional Review Board of MBRU (MBRU-IRB-2023-246), the MCME Research and Ethics Committee (MCME.CR.338.MCIT.2024), and Dubai Scientific Research and Ethics Committee (DSREC) at the DHA (DSREC-GL05-2024).

### Data collection

Participants [i.e., Key Opinion Leaders (KOLs)] were given the option of providing their feedback either in writing, using the questionnaire, or in the form of a semi-structured interview. In both cases, the same tailor-made survey/ interview protocol was utilized (Appendix 1). The protocol was composed of five segments: the first segment inquired about the trigger that initiated the respective trajectory; the second segment solicited assessments and reflections around the situation back then; the third segment inquired for feedback on the results of the affiliation formation; the fourth segment queried about the nature of the relationship between both entities; and the last segment involved creating the space for further reflections. Prior to deployment, the interview protocol underwent two validation phases. Firstly, three subject matter experts were engaged in the content validity. Secondly, the questions of the generated tool were discussed with a middle manager at MBRU (who was neither directly nor indirectly involved in the affiliation reported upon in the current study) to assess the clarity, readability, and comprehensibility of the questions, and the flow by which they are presented (i.e., face validity).

The study's participants included all the affiliation's primary stakeholders. According to the corresponding theory, “stakeholders” represent anyone with an interest in the respective endeavor (70). The term was first defined as follows: any group or individual who can affect or is affected by the achievement of the organization's objectives (71). At a later point in time, it was stated that stakeholders are the individuals who are considered to be vital to the sustainability and in turn success of the endeavor (72). Primary and secondary stakeholders represent the differing stakeholder levels which can exist within any one network/ system. The continued participation of a primary stakeholder is essential to the corporation's survival, while secondary stakeholders are those who, despite their influence on or by the organization, are not essential for its continued existence (73). Accordingly, in the context of the current study, all stakeholders relevant to the respective public private affiliation were mapped onto concentric circles representing stakeholder levels, with the innermost circle representing the primary level, and subsequent circles expanding outward to represent less relevant levels. All the stakeholders positioned in the innermost circle were invited to participate in the current study. These constituted of a total of 18 KOLs were ones who played an active role in developing the respective affiliation (2 were female and the rest were male, and 3 were UAE nationals and the rest were from the following countries, listed in alphabetical order: Canada, Egypt, Nigeria, South Africa, Sudan, Syria, United Kingdom, and United States of America). Those KOLs (at the time of affiliation) were senior leaders, managers, academic leads, faculty members, and clinicians, some of whom were concurrently handling more than one role. The research participants gave their verbal and/ or written informed consent prior to participation (i.e., filling in the survey or the initiation of the interview). At the start of each interview session, the interviewer reassured the participants of the data confidentiality and anonymity, and built rapport with them. Participants were encouraged to share any ideas and thoughts that surfaced for them as the conversation unfolded. The interviewers focused on holding space for the participants which allowed for substantial reflectivity. In addition, as per the guidelines of the participant-focused qualitative research, the interviewers were aware of what personal characteristics (e.g., prior experience and beliefs) influence their subjectivity, and remained mindful about withholding their opinions while conducting the sessions to avoid data contamination.

All interviews were set down in writing and/ or recorded. The recordings underwent verbatim transcription by one member of the research team. These transcripts, along with the data collected via the questionnaire, constituted the dataset which was systematically analyzed for the purpose of the current research. To protect the anonymity of the participants, each participant was assigned a unique identifier, composed of three parts: a serial number (i.e., 01 through 18), followed by “M” for Male or “F” for Female, and then, “MB” for MBRU, “MC” for MCME, or “MCI” for MCI. If the participant had, at the time of affiliation, roles in both MBRU and MCME, the third part of the identifier was “MBMC”. For example, the identifier: 16-M-MBMC, represents participant number 16, who is a male, and who, at the time of affiliation, had roles in both MBRU and MCME.

### Data analysis

The data analysis started after the completion of the data collection phase. The data was inductively analyzed, in an iterative manner and based upon constructivist epistemology (69). This was done using a participant-focused, phenomenological approach to inductive thematic analysis by two researchers (L.D. and F.O.). The researchers recognized upfront the factors that could influence their perceptions regarding the subject matter. Consistency, regarding the underlying assumptions and theories, was assured throughout the study (by one of the two data analyzers who has developed, over time, expertise in inductive, qualitative socio-behavioral research). By embracing rather than avoiding the researchers' personal involvement in the investigation and by evaluating interpretations according to their impact on readers, investigators, and participants (74), the quality control attained in this investigation shifted from the objective truth of statements to understanding by people. This interpretive approach to research is different than conventional scientific inquiry as it involves the ability to recognize and recreate the experiences of the participants. The target of this approach is to understand the participating human beings, and their thoughts and ideas, and motives, aspirations, and actions, rather than to find casual explanations. This methodology assumes that we can explain human beings' thoughts and emotions by actively listening to and understanding what they are saying/their self-expressions.

The qualitative analysis process, adapted for this study, followed the six-step framework initially introduced in 2006 by Braun and Clarke (65). This multi-phased approach to inductive thematic analysis is widespread and has been repetitively used in scientifically-sound knowledge sharing activities within the context of the research study (28, 75). NVivo software ver. 12.0 plus (QSR International Pty. Ltd., Chadstone, Australia) was used to code the data, and in turn facilitate the categorization of the identified text fragments.

**Figure 3:**
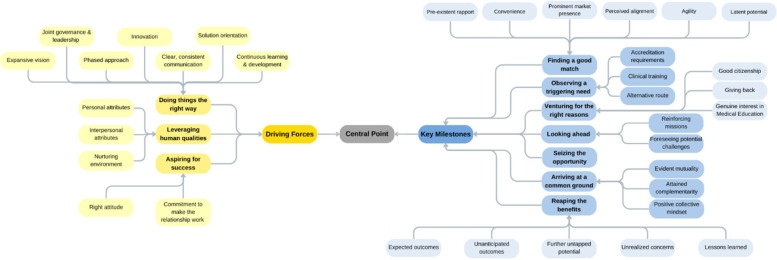
Mind map deployed as a tool to facilitate the qualitative analysis.

The analysis process started with the two researchers (L.D. and F.O.) familiarizing themselves with the compiled dataset. They read through the dataset together, thoroughly reflecting upon the content of the deidentified data. The second step of the analysis process consisted of reviewing the transcripts while extracting the text fragments that relate, directly or indirectly, to the preset research question. As such, any text segment relating to the journey by which the affiliation between the public medical school and the private healthcare provider occurred got tagged. This kept going until data saturation was attained (i.e., no new information/ insight was observed in the datasets). This systematic review led to the generation of categories of text fragments which set the stage for the researchers to work on the third step of analysis. These categories underwent several rounds of reflections; the different ways by which these categories could relate to one another were identified (leading to several potential interconnections). In the fourth step of the qualitative analysis, the categories of text fragments were brought together to form higher-order themes, according to the interconnection that made most sense to the two researchers (Figure 3).

All the categories and themes were then coded (i.e., given a label/title) and defined, in the context of the study, to complete the fifth step of the analysis. The output of this step constituted the study's conceptual framework which guided the last step of the multi-staged inductive thematic analysis: reporting upon the findings, which was done narratively in alignment with established guidelines, including the Standards for Reporting Qualitative Research (SRQR) (76–79). To further corroborate the findings, the researchers generated a tally and reported on the number of text fragments within each category, within the identified themes. If for a single participant, more than one relevant text fragment was identified within the same category, they were all collectively considered as one entry. Accordingly, the tally reflects the number of participants that brought up matters relevant to each of the respective categories.

After the completion of step five (i.e., development of the conceptual framework) and prior to step six (i.e., the reporting narratively on the results of the analysis), a respondent validation was conducted. The informant feedback of all the participants was obtained through a virtual meeting. This study's principal investigator showed the participants three PowerPoint Presentation slides. These slides included the research questions, a brief explanation of the adapted process of qualitative analysis, and the study's conceptual framework. The participants were given the opportunity to share their reflections regarding the extent of resonance between their responses to the interview/ survey and the generated conceptual framework. The meeting attendees agreed with the identified codes, and how the generated conceptual framework portrays the sequence of events and identified moderators.

## Results

**Figure 4:**
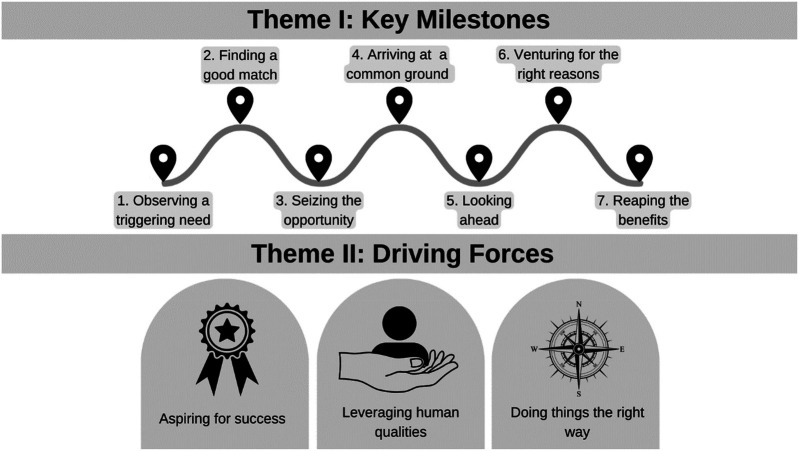
The study's conceptual framework: “public private affiliation journey”.

The qualitative analysis led, as per this study's conceptual framework: “Public Private Affiliation Journey” (Figure 4), to two interconnected themes, namely: Key Milestones and Driving Forces.

Within Key Milestones, seven sequential categories were identified: Observing a triggering need, Finding a good match, Seizing the opportunity, Arriving at a common ground, Looking ahead, Venturing for the right reasons, and Reaping the benefits. Within the second theme: Driving Forces, the following three categories were identified: Aspiring for success, Leveraging human qualities, and Doing things the right way.

The tally of text fragments showed the distribution, outline in Table 1.

**Table 1:** Semi-quantitative tally of the output of the participant-focused qualitative analysis.

Theme	Key Milestones							Driving Forces		
Category	1	2	3	4	5	6	7	Aspiring for Success	Leveraging human qualities	Doing things the right way
Tally of participants (out of 18)	6	13	16	16	13	9	13	12	14	15

### Key milestones

This theme encapsulated the segments of the text that relate to the participants' perception of the steps that the involved parties collectively needed to take to progress in the public private affiliation journey referred to in the current study.

#### Observing a triggering need

The first category of this theme included quotes that reveal what participants consider as the starting point of the respective journey.

15-M-MBMC: “…The affiliation was born out of necessity…”

The journey, in its entirety, began with an evident need at the public medical school side.

1-M-MB: “…MBRU, as a medical school, needed a teaching hospital…”

6-M-MC: “…they wanted to establish a medical school. They needed a clinical partner for the placements…”

It seems, from the participants' reflections, that there had been an initial plan that did not get realized, which is why the involved parties of the public medical school needed to explore alternative paths.

1-M-MB: “…the initial plan, years before the inception of the University, was for MBRU to have its own hospital; this was put on hold.”

8-M-MC: “…my understanding is that previously a 400-bed University Hospital was planned for MBRU. This project was delayed meaning that the University needed a trusted clinical partner that could fulfil this role…”

Given the determination not to modify the initial timeline set in place, the involved parties of the public university had to decide and act swiftly.

3-M-MB: “…there was a pressing need to find an alternative…”

15-M-MBMC: “…We needed to collaborate quickly with an entity to enable learning in the clinical environment…”

They also needed to live up to institutional licensing and program accreditation requirements, where the UAE higher education regulator requires (rightfully so) for any medical school to have secured one or more sites for clinical placements prior to launching the medical program.

1-M-MB: “..the initial trigger was that MBRU needed to collaborate with healthcare provider(s), to start with, for institutional accreditation and also for the MBBS accreditation [by the CAA]…”

#### Finding a good match

The second category of this theme included participants' personal reflections and opinions on the differing variables that they believe the involved parties of the public university considered when selecting the respective private healthcare provider as “the most suitable partner”.

Although the university was public and the healthcare provider was private, there appeared to be more important variables upon which the public university party based their decision.

To start with, there was a clear element of practicality. According to the study's participants, the involved parties valued the fact that the location of the primary hospital for the clinical placements was literally across the road from the medical school.

8-M-MC: “…Mediclinic City Hospital is also ideally located right opposite to MBRU…”

17-M-MBMC: “…Mediclinic City Hospital was quite a convenient option as a teaching hospital since it is across the street from MBRU. However, it is worth noting that there were other hospitals in proximity to MBRU, which were not considered for partnership…”

Apparently, the involved parties also took into consideration regulatory aspects. They believed that the fact that both institutions: the medical school and the selected private healthcare provider, belong to the same jurisdiction is likely to ease the entailed processes and needed clearances.

3-M-MB: “…Being part of the same licensing jurisdiction (i.e., DHCC freezone), which was an advantage back then…”

6-M-MC: “…Initially, from MCME side, it was just Mediclinic City Hospital, which means both the hospital and university were in DHCC which made clearing the matter from a regulation’s perspective quite straight-forward. Later, as more facilities became part of the equation, DHA jurisdiction got incorporated. By then, we had a clear understanding of what we are after and how to go about it…”

The study participants also highlighted that the preexistent rapport between the involved parties and between the respective institutions enabled the relationship. There seemed to have been positive preconceptions, and clear willingness to collaborate and co-create, on both sides.

1-M-MB: “…our existent rapport with the leadership, mainly the Senior Corporate Medical Director and Chief Operating Officer…I had some previous work experience with MCME key stakeholders, where I contributed to developing continuing education programs…there was a Memorandum of Understanding (MoU), as a foundation to explore opportunities for collaboration, between MCME and Mohammed Bin Rashid Academic Medical Center (the institution which predated MBRU)…”

6-M-MC: “…There was also a rapport between both institutions. The key stakeholders of both institutions knew each other. MBRU was well aware of MCME and what it stands for; they would not have approached an organization that they knew nothing about…”

Moreover, the participants also reflected on the prominent market presence of the selected healthcare provider. It seems that this variable, as well, was taken into account by MBRU.

15-M-MBMC: “…MCME is a major healthcare provider, in the private sector …Afterall, MCME is the largest private sector provider in UAE…”

17-M-MBMC: “…MCME is an established healthcare system in UAE…”

The respective healthcare provider had a strong societal reputation and significant market coverage.

1-M-MB: “…we wanted to become partners with MCME because of its societal reputation…MCME is international, covers almost all disciplines, and has substantial outreach…”

6-M-MC: “…So, in summary, I think MBRU chose MCME because it was well-established and has a good reputation…what attracted them to MCME is, in my opinion, that it is well-established, credible..MCME is actually a leader in the private healthcare sector in Dubai…”

Another variable was the extent of perceived alignment between both institutions and also among the involved parties.

18-F-MBMC: “…the value proposition of MCME suffices to understand why it was selected: ‘…a well-established private healthcare system with high standards of clinical excellence, quality, and patient safety…’. Educating medical students in such an environment is beneficial to their learning and will positively shape their future practice…”

Both institutions were considered by the study's participants to be characterized by value-based cultures, where many of the institutional resources are directed towards attaining and maintaining the quality of the environment.

6-M-MC: “…and it is clearly a value-based institution …”

8-M-MC: “…MCME had considered collaborating with other universities. Yet, MBRU, with the name of the ruler of Dubai as its name, appeared as the only match, with values aligned with those of MCME…MCI is an international healthcare company known for its ethical values and clinical excellence, and as such was considered the ‘perfect choice’…”

Also, according to the study's participants, the involved parties at the medical school side perceived latent potential that they believed was worth realizing at the private healthcare provider's side.

7-M-MC: “…and quite possibly the CoM Founding Dean’s awareness of MCME’s potential…”

Lastly, given the criticality of the time factor, the involved parties at the medical school side needed to identify a partner that is agile in terms of taking decisions and also in terms of implementation.

10-F-MC: “…MCME…credible human capital and effective operational framework which MBRU leveraged upon…”

15-M-MBMC: “…we had very little options for potential partners who are sufficiently agile. We found what we need in MCME leadership…is responsive and reliable…MCME swiftly provided a lot of the needed resources in the clinical environment [e.g., student-specific access to Electronic Medical Records (EMR), meeting rooms, and lounge access] which enabled effective students’ integration into the MCME system…”

#### Seizing the opportunity

The third category, within this theme, related to the study participants' insights about the perceived potential inherent in the prospective collaboration and how the involved parties went about embracing the affiliation proposal.

1-M-MB: “…our academic health system was missing a component, and MBRU and MCME collaboratively created this affiliation opportunity and seized it…we were pioneers. Since then, public private partnerships have been becoming more and more common in the UAE, not only in the education/ medical realm…”

2-M-MB: “…It is a road that has not been travelled on before in this Emirate…”

The participants kept bringing up how the private healthcare provider responded positively.

13-M-MC: “…To contribute to the establishment of a new medical school from the beginning, it has been exciting and a privilege…”

There was also protectiveness of the relationship (at the private healthcare provider's side), especially when matters started taking shape and in turn the involved parties became more aware of the true value of what was happening.

6-M-MC: “…A perceived threat back then made MCME quite protective of their relationship with MBRU. The whole healthcare sector was discussing this affiliation, and the other private sector hospitals were keen to come into the picture. We wanted everything to be seamless and to exhibit credibility in rising in order to the challenge to gain the trust of MBRU leaders. We felt we were obliged to excel to maintain the relationship with MBRU…”

It appeared that the involved parties were well aware of the novelty of the situation, and the collective eagerness to become pioneers was evident to the study's participants. It seemed to the study's participants that the involved parties considered this venture as an opportunity to do something unique; to “leave one's mark”.

4-M-MB: “…MBRU approached an operator in the private sector that never trained medical students before. MBRU became the first medical school in Dubai to expose medical students to patients who seek care in the private sector (today, 70% of the MBBS clinical placements are in a single private operator)…”

6-M-MC: “…the affiliation was unique; there was no existent system or model out there that we could have followed…MCME believed it was a wonderful opportunity to do something a little bit different…I would go all the way to say everything about this affiliation is unique. A public medical school decided to affiliate itself with a private sector healthcare provider as opposed to the public sector. This is unusual…”

The participants believed that the involved parties chose to challenge preconceived notions, because they saw the inherent potential of the private sector and the unique advantages it can offer.

3-M-MB: “…Opening one’s mind about the potential contribution of the private sector to teaching…debunking the myth that private practice medicine is only for money, and that clinical training of doctors can only succeed in public environments…”

15-M-MBMC: “…some of MBRU founding faculty members- including myself …who were trained in academic health systems (typically- not-for-profit, public sector), had concerns about how collaborating with a ‘for-profit, private’ institution will affect the quality of education…Generally speaking, there is a perception that physicians who work in the private sector tend to be driven by profit generation, which differs from the primary motives of those who work in the public sector…we learned not to limit our understanding of institutions to their labels: ‘public/ private’ and ‘for profit/ not for profit’…”

According to the participants, all the involved parties wanted to realize the potential of the private sector.

1-M-MB: “…This affiliation realized a nascent opportunity for MCME, enabling it to go to the next level of care…We learned that just because a healthcare provider operates in the private sector does not make it unsuitable for education. Our experience shows that private providers can contribute to health professionals learning and teaching…”

10-F-MC: “…another motivator for MCME to collaborate with MBRU was the obvious latent potential of MCME to be at the forefront in clinical medical education in the region, as private sector participation is becoming more common…”

According to the study's participants, a clear landmark in the affiliation journey was when a shared, informed decision was made by both parties.

1-M-MB: “…there was clear willingness to collaborate and co-create, along with the ’seriousness’, exhibited as responsiveness, reliability, and robustness…MCME were excited to work with MBRU leadership…”

17-M-MBMC: “…as a young academic institution, carrying the name of the ruler of Dubai (His Highness Sheikh Mohammed bin Rashid Al Maktoum) made it an excellent opportunity to collaborate…”

To the participants, the involved parties perceived the configuration to be logical and feasible.

8-M-MC: “…when we opened Mediclinic City Hospital (i.e., our flagship facility), we deliberately offered the most basic of healthcare services at the outset but gradually increased the clinical complexity…As we eventually became a more tertiary level hospital that people trusted, the next logical step was to introduce research and to become a teaching hospital which would further enhance the trust of all stakeholders in the MCME brand…”

10-F-MC: “…the public private partnership provides a logical solution for MBRU in managing resources (financial or otherwise) towards establishing medical education programs and frameworks…”

Yet, the involved parties, according to the study's participants, were in full realization of the entailed uncertainty and needed to *assume* trust at the beginning.

6-M-MC: “…we needed to think out-of-the-box, experiment, take risks. We had to trust each other; at the beginning, there was a level of naivety… It turned out that both sides were up to the trust…”

According to the study's participants, the involved parties approached the matter experimentally, taking calculated risks (given that the stakes were high), and deploying entrepreneurial thinking.

3-M-MB: “…highly successful ‘experiment’ with improvements in care, research, and education. A very good relationship developed with time…”

6-M-MC: “…we needed to think out-of-the-box, experiment, take risks…We were not doing something that was done before. If we had applied a preexisting model (i.e., a framework that proved effective elsewhere), we would have been more confident in terms of planning; there would have been concrete steps with proper change management. We would not approach it as experimentation…”

#### Arriving at a common ground

This category referred to the text fragments relating to a discrete step where a consensus was built among the involved parties.

8-M-MC: “…we became an integral part of the team at MBRU and saw ourselves as one…Fortunately, MCME leadership teams, especially the MCI Chairman, became very supportive of this initiative, soon after the strategy took shape…”

17-M-MBMC: “…A very thoughtful arrangement was agreed upon between the two institutions to have specific joint appointments to support the collaboration…”

This included, according to the study's participants, leveraging clear commonalities, along with proactively nurturing a collective mindset.

2-M-MB: “…The interest expressed by the senior leadership of MCME in education and research…Mutual interest in developing the next generation of physicians …”

13-M-MC: “…MBRU and MCME have shared values and goals; both are strategic players in the market and are committed to contribute to realizing the vision of UAE…Both parties believe in the three key pillars of the relationship- medical education, clinical practice, and research…”

The study's participants seemed to believe that there had been evident mutuality thus far, in terms of what both parties stand for and how they go about matters, and also in terms of future aspirations and strategic benefits. The mutual trust and respect among both parties were clear to the participants. This win-win configuration, according to the participants, is what made the relationship sustainable over time.

5-M-MB: “…Aligned visions and ethos at leadership level…”

11-M-MC: “…A shared vision, common purpose…”

These similarities, according to the study's participants, made the affiliation easier.

3-M-MB: “…Assuring mutual benefits…MCME always wanted to affiliate itself with UAE institutions…MBRU has features of private sector which encouraged the affiliation…trust, common objective…we were ‘equals’ in the pursuit of the common good; both institutions benefited from the affiliation…”

15-M-MBMC: “…the Founding Chairman of MCI was supportive and interested in the affiliation; the concept was not totally foreign to MCI…”

Also, the complementarity between the institutions, in terms of capabilities and resources, was acknowledged by the study's participants.

2-M-MB: “…The agreement required MBRU to develop the curriculum and teaching schedule, and MCME to deliver and assess the students’ performance. As such, it proved to be important for MCME to be well involved in developing the curriculum…”

10-F-MC: “…The public private partnership provided MBRU with access and opportunity to utilize the diverse valuable resources (predominantly human capital) that MCME has…MCME benefitted from the medical research opportunities and collaboration which is believed to improve healthcare outcomes. It may also reduce health costs in the long-term…”

There was clearly a positive, collective mindset, where everyone valued the entailed opportunities.

2-M-MB: “…MBRU noted interest of physicians at MCME in teaching and starting/ maintaining academic tenure as Adjunct Faculty…The belief that involvement in academia improves the quality of service by providing opportunity to continuously improve oneself and to be a role model…”

4-M-MB: “…people believing that the practice of up-to-date medicine and education cannot be separated…”

Several participants highlighted that there could have been more work done to get the support and buy-in of the physicians.

7-M-MC: “…Make sure, as much as possible, that you have all physician groups along for the ride…”

11-M-MC: “…Engage doctors more at the beginning to make the process more doctor-driven rather than management-driven…Proactively managing doctor’s expectations regarding compensating for teaching…”

The participants also believed that the collective mindset is actually what enabled the relationship to withstand the unprecedented test of the pandemic.

15-M-MBMC: “…COVID-19 tested the relationship between MBRU and MCME. The affiliation stood the test of those exceptionally challenging times. MCME never rejected the students. It was MBRU’s decision to temporarily pull out the students from the placements, given the concerns about their health and wellbeing. After a short while, we collectively decided to resume the rotations, with all the necessary precautions provided by MCME. This turned out to be the right decision, after all…”

#### Looking ahead

This category shed light on what the involved parties defined as the goals, from the point of view of the participants. It seems that the affiliation between the involved parties began with the end in mind. The desired destination was clearly defined up-front. Involved parties were driven because they believed that the affiliation would lead to reinforcing the goals of both entities; participating in this journey was considered, by the involved parties: a way of investing in the future.

7-M-MC: “…Becoming a teaching hospital changes the institution for the better. The academic mindset is sharper, and the students keep you on your toes! This in turn tends to attract better quality medical staff, going forward. Patients tend to assume that if we have medical students then we must be good..”

9-M-MC: “…the affiliation originated from the need to offer high-quality medical education to provide, in the future, healthcare services to the local community…It was believed that by forming an affiliation with MBRU, MCME will gain numerous benefits, including but not limited to: access to highly trained medical personnel, advancement in medical research, reputation enhancement, and improved patient outcomes. It was believed that the affiliation could support MCME in attracting and training a new generation of highly qualified medical professionals…”

Both parties wanted to invest in the future by creating a pipeline for future physicians. This was believed to address the growing demand for advanced medical services in the region. Integrating existing entities to create “a whole that is more than the sum of its parts” was on the horizon.

11-M-MC: “…MCME was motivated to affiliate itself with MBRU to differentiate itself as an academic health system; to make an impact and contribution to the community; to enhance doctor’s practice through continued professional development integral to fulfilling teaching requirements [where MBRU introduced the ACE for the adjunct faculty]…”

Interestingly, the participants highlighted that the involved parties, back then, foresaw challenges, which enabled them to address them head on.

13-M-MC: “…The success factors include…acknowledgement of challenges, early on, during the process…”

18-F-MBMC: “…The challenge was the shift in mindset about ‘paying for services’ evolving into a more humanitarian vision, and instead jointly contributing to a higher cause…”

According to the participants, the involved parties were cognizant of all the possible way in which the affiliation could affect the different aspects of the quality of care, including safety, patient-centeredness, effectiveness and efficiency, timeliness, and access and equity.

10-F-MC: “…disruption in MCME operational framework and workflows to accommodate medical students..Potential impact on MCME’s financial and technical capacity. Appropriate and effective communication of value proposition of public private partnership to the relevant stakeholders and players…”

The forecasted challenges and risks mentioned by the participants included accommodating and integrating the students into the existing healthcare delivery system.

2-M-MB: “… we were thinking that the private sector may not be accommodative of large groups of students, MCME may not be interested in investing in educational resources (e.g., journals, onsite reference texts, and on-call rooms), and MCME staff may be resistant to effectively integrate trainees into the healthcare teams…”

4-M-MB: “…For the learners, the challenge was to develop the confidence to interact and learn from observing and/ or interacting with patients (this is especially relevant to those who seek private care, those tend to have greater expectations)…”

The participants also reflected on the difficulties related to assigning academic responsibilities to clinicians, where there were concerns around productivity, competence, and managing expectations.

14-M-MCI: “…These were some of the concerns that we had: would enough MCME doctors be willing to make some of their time available for the training of the students? how would they be compensated? how would the doctors’ medical malpractice insurance handle potential claims?..”

15-M-MBMC: “…another concern was: how do we get the adjunct faculty up-to-speed?…”

Assuring the quality of the education was also brought up by the study's participants.

10-F-MC: “…An additional specific challenge the partnership had to navigate was assuring the quality (i.e., content, richness, credibility, and effectiveness) of the curriculums and educational programs. This required taking into consideration the teaching styles and modes of education delivery, the recruitment and performance appraisal of the educators (at MBRU and at MCME facilities), and the accreditation of the educational programs and training facilities…”

Lastly, the involved parties were concerned about patients' acceptance.

2-M-MB: “…there was a concern that patients in the private sector may not welcome trainees…”

#### Venturing for the right reasons

This category included all the text fragments which show that the participants believe that the involved parties embarked on this journey with a clear “why” and that these reasons were entrenched in benevolence and social responsibility.

1-M-MB: “…MCME foresaw the value of this journey and chose to embark on it without any external (financial) incentives (such as those offered to academic hospitals in the western world). MCME wanted it to happen…”

13-M-MC: “…Trust, ethical approach, mutual respect, long-term strategy, commitment to teaching and contribution to the realization of the UAE vision, and strong and committed leadership from MBRU and MCME…it reinforced our commitment to teaching and research…”

To the participants, there were pure intentions and good citizenship among the involved parties, where efforts were aligned with the local and federal direction. At some point, the financial element, all together, was pushed aside. All involved parties appeared to the participants to be altruistic, after a higher cause.

11-M-MC: “…The initial financial agreement never got implemented. The fact that this did not compromise the relationship is, in of itself, reflective of the quality and strength of the relationship…”

15-M-MBMC: “…yes, we signed an agreement as a formality. In effect, it was not required. What actually took place was more like a ‘gentlemen’s agreement’, sealed with a ‘handshake’; the financials (eventually) were left out. The financial elements of the agreement, whereby MBRU would remunerate MCME for the time doctors spend teaching students, were never implemented…”

The involved parties wanted to give back to the community-at-large.

8-M-MC: “…it gave us the opportunity to support Dubai leadership in fulfilling their objective of providing an international university to the People of Dubai, we wanted to be part of that journey as a way to thank the leadership for their trust in us…”

9-M-MC: “…the affiliation reflected the broader trend of public private partnerships in the healthcare sector, aimed at addressing the growing demand for advanced medical services in the region…”

There was genuine interest in health professions education and how this ultimately leads to better outcomes of care.

6-M-MC: “…We collectively believed that it would create a positive impact on patient care and patient experience…It gave us a favorable presence in the market; a lot of physicians were attracted to work at MCME because they get to exercise their passion for academia, giveback to the community, teach medical students…”

16-M-MBMC: “…the most important factor was the interest in and commitment to medical education of the Chairman of MCI. He has always been interested in medical education and managed to instill that at the other hospitals in South Africa…”

#### Reaping the (immediate) benefits

This category revolved around the participants input in regard to what they perceived as the immediate benefits of the affiliation.

6-M-MC: “…The primary benefit to MBRU is definitely the clinical placements…The effect was mostly positive. Having students from MBRU gave MCME a great sense of pride. With time, we enabled the placement of students across many clinics. The rate of accepting medical students among patients was really high. MCME were speaking of this affiliation everywhere. The chairman was thrilled. There was tremendous institutional pride…”

9-M-MC: “…through the affiliation with MCME, MBRU enabled its students to receive training in up-to-date medical treatments, which may lead to improved patient care…”

Apparently, some of these benefits were anticipated, such as the fulfillment of preset objectives around clinical teaching. This came together with a sense of pride.

10-F-MC: “…the affiliation enabled us to become a strategic partner for a governmental entity (i.e., Dubai Health) and a significant contributor in establishing health professions education/training in the region. It also made recognizing MCME institutions as training facilities possible (by regulators and accreditation bodies) …”

15-M-MBMC: “…The immediate output was successfully starting placements for the medical students in surgery, internal medicine, pediatrics, and family medicine…Also, the affiliation, in general, and specifically the clinical learning environment met the CAA requirements…The fact that it is a success is a no-brainer…In terms of outcomes, we are getting quite positive feedback about the MBRU MBBS graduates’ clinical performance in residency, across disciplines. The MBBS graduates’ performance in Emirates Medical Residency Entrance Examination (EMREE) and United States Medical Licensing Examination (USMLE), including STEP 2 (clinical), is indicative of the effectiveness of the clinical placements integral to MBRU’s MBBS…”

Other outputs were unexpected, appearing as byproducts to the study's participants.

1-M-MB: “…the affiliation went way beyond its initial scope. The kidney transplant program is (in of itself) a huge success story that was born out of this affiliation. A lot of lives have been saved (to date) because of this transplant program that stemmed from collaborations between clinical faculty from MBRU and stakeholders from MCME…You can also look into the number of published peer-reviewed articles that are based on research collaborations between MBRU and MCME- all these were byproducts of the agreement…The clinicians benefitted from this affiliation, especially those who were used to working in academia. They got academic titles. This is an advantage of working in an academic health system, such as: Dubai Health; it attracts and retains health professionals… as matters progressed, we developed several contractual models to attract competent professionals, offering them a dual role, as clinicians and faculty…”

8-M-MC: “…The affiliation also gave us the opportunity to attract clinicians who were interested in teaching and research. Ordinarily, this type of clinicians tends to continuously learn, and as such offer a superior, up-to-date service to patients (further enhancing the trust in MCME brand)…”

To the study participants, there was realization back then that there is further untapped potential that they were eager to realize. This was forming a source of renewable energy.

13-M-MC: “…The relationship between both entities still has plenty of opportunities to further develop and expand to include postgraduate medical education, research, innovation, dentistry, nursing, and continuous professional development…The affiliation enabled attracting the right type of doctor that is committed to teaching and research…”

Also, it was clear to the participants that fortunately some of the initial concerns did not get realized.

13-M-MC: “…There was the potential of negative impact on the clinical productivity of the doctors due to teaching commitments. Interestingly, no significant impact was noted, and students’ presence actually added value to patient-physician encounters…”

The participants thoroughly reflected upon lessons learned and how both institutions organically evolved.

1-M-MB: “…we learned from MCME, when it came to the governance and organization that happened around the strategic decisions integral to the affiliation …”

6-M-MC: “…I think it would have been useful to proactively address the resistance among some of the physicians. Those minority who believed they are supposed to be reimbursed for their teaching and who were convinced that MCME was getting paid by MBRU. It was like a rumor in MCME among the physicians and it took us time to fully dissipate it …”

9-M-MC: “…we learned the importance of balancing clinical education and patient care. The affiliation has certainly highlighted the importance of balancing the needs of medical students and of patients, and how both parties can work together to achieve this balance…”

### Driving forces

This theme encapsulated the text segments of the transcripts that relate to the participants' perception of what enabled progression in the steps of the public private affiliation journey.

#### Aspiring for success

The participants seemed to believe that the collective aspiration for success was catalyzing the situation, where the involved parties were clearly “in it to win it”.

6-M-MC: “…The reality is everybody just thought it was a great idea! This was the overall sense of the situation…the risk was managed not through governance but through commitment to the relationship…”

The involved parties, according to the participants, were committed to making the relationship work. They really wanted it to happen.

13-M-MC: “…Relationship based on trust. Absolute commitment by both parties…”

18-F-MBMC: “…it is a relationship born out of mutual respect and trust, which translates into leaders and employees who are enthusiastic and committed to making the relationship work…”

They had the “right attitude”, which was the case even when they faced challenges.

7-M-MC: “…our MCME leadership recognized upfront the potential and the responsibility of becoming involved…”

16-M-MBMC: “…MCME gave MBRU a reliable site to place students during their clinical training years. Generally, there was enthusiasm on both sides, and this greatly assisted the success for the first cohorts of MBRU students…”

This seemed to have led to a ‘ripple effect”, where the right attitude appeared to be contagious among the involved parties.

1-M-MB: “…We eventually created a common brand. No one made a big fuss about the affiliation, went around advertising/ marketing for it, which was particularly unique for a private healthcare provider…”

3-M-MB: “…a great deal of flexibility was introduced; assigning adjunct faculty with academic titles facilitated cooperation with clinical staff; no one took any feedback personally… students got integrated within MCME, and healthcare may have improved as a consequence to the physicians stepping-up to fulfilling their teaching responsibilities…”

#### Leveraging human qualities

The participants seemed to believe that human qualities were effectively leveraged throughout the journey. The confidence that they had with the credentials and credibility of all the involved parties was frequently alluded to.

13-M-MC: “…Dedicated people (e.g., discipline leads, academic coordinators, and director of academic affairs) and clear responsibilities (e.g., joint appointment of director of academic affairs)…The success factors include… committed leadership…I knew that such a public private partnership would not just be a smooth road to travel on, but I had confidence that with the attitude and the ability of the people involved any unexpected obstacle would not be insurmountable…”

A lot of what the participants referred to were personal attributes of the involved parties. This includes the prominent goodwill of the involved parties.

8-M-MC: “…I believe the key success factors are first and foremost ‘trust’, but also transparency and the commitment to a common goal…”

14-M-MCI: “…this public private partnership seems to be a real success… I believe it had a lot to do with … the competence, integrity, and tenacity of the people involved…”

Leadership traits were also repetitively alluded to by the participants.

4-M-MB: “…Highly professional leadership at both institutions…Good intentions work really well, especially if complemented with a good mix of experience and exposure to other systems…It was something new and as the level of success became obvious to the two institutions, the level of commitment increased…”

Apparently, there was at some point concern about the potential overreliance on specific individuals.

6-M-MC: “…we are yet to solidify all the systems and processes around the governance structure. The human factor is very strong… Yet, if a change in management occurred (on either side), this may shake the affiliation. There is reliance on a selection of the key leaders, which in fact contributed to the success of the affiliation… working on reinforcing existing systems would safeguard the affiliation in the long-run and will maximize the value for all involved stakeholders…”

15-M-MBMC: “…It was obvious, from the beginning, that the affiliation was effective, but then the question became: how do we sustain this? The affiliation, at the very beginning, was highly dependent on the leadership in both entities, and this constituted a concern…”

The study participants frequently alluded to the attitudinal shift that needed to take place among the physicians.

17-M-MBMC: “…Many physicians resisted getting involved in student education citing competing responsibilities (where teaching was believed to require additional time of physicians in their clinics and wards) and the potential discomfort/ refusal of patients to have students around, which could have impacted the flow of patient care and revenues. This issue created, at some units, unease between MCME management and its physicians. The management took a firm stance and maintained an unwavering commitment to the relationship between MBRU and MCME hospitals…”

There were also a lot of interpersonal attributes that the participants elaborated upon.

6-M-MC: “…I think at the core of all good collaborations there are healthy personal relations. The relationship between MBRU senior leadership, especially the Founding Dean of College of Medicine and the Vice Chancellor at MBRU, and MCME was very strong. These strong personal relationships have been key to the sustainability of the affiliation…”

Some were referring to relationships within the same institution, and others were considering human connections between the two institutions.

13-M-MC: “… Joint committees with clear Terms of Reference and balanced representation from both parties…”

16-M-MBMC: “…Quality of relationships, commitment, and values trickle-down by the top leaders in both institutions…”

The human qualities were nurtured through the environment.

13-M-MC: “…and recognition of successes…There is a joint initiative between MBRU-MCME underway to recognize the doctors’ teaching (by way of an award ceremony) …”

18-F-MBMC: “…Mutual respect, transparency, and the continuous acknowledgement from the Vice Chancellor at MBRU about the role MCME plays in MBRU as a valued clinical partner…The need to acknowledge those making active contributions to teaching in the clinical environment and continuously support each other, to shout out even the smallest ‘wins’…”

If it was not for the limited time, the involved parties would have liked to invest more in getting the buy-in of the physicians.

15-M-MBMC: “…One thing maybe we could have done differently, if we had the luxury of time, is to better socialize the idea among the Adjunct Faculty. We did not have that option, though…”

#### Doing things the right way

A particular modus operandi, characteristic of the affiliation reported upon in the current study, seemed to organically arise as matters were unfolding, and this specific “way of doing things” became the engine that was transforming the involved parties' aspirations to reality.

10-F-MC: “…The necessity to have a defined legal, contractual, and governance framework for public private partnership that provides clarity on the roles and responsibilities of both MBRU and MCME in the public private partnership agreement. This agreement should bear consideration of pertinent factors like risk sharing and management, appropriate utilization of resources, MCME’s financial and technical capacity to shoulder this agreement as well as both parties’ commitment to public private partnership…”

17-M-MBMC: “…Persistence in achieving the goals, having clear objectives to resort to when dealing with obstacles, and continuously and clearly reiterating the goals and objectives to medical staff and faculty…”

A lot of the text fragments, from the transcripts, were related to the participants' reflections on the involved parties' expansive vision. The participants saw that the vision set in place, reflected in the agreement and the corresponding planning, paved the way for the journey.

9-M-MC: “… A comprehensive and well-crafted affiliation agreement that outlines the terms and responsibilities of each party…”

11-M-MC: “…protective factors against obstacles include long-term commitment by MCME, where the affiliation was set out to be renewed after 3 years…”

In some cases, the participants highlighted how the involved parties could have further modified the plans set in place to account for the change that was underway.

11-M-MC: “…Since Parkview Hospital was not built yet, at the beginning of the affiliation, and we needed to adapt Mediclinic City Hospital for teaching, it would have been beneficial to take into account the academic activity in the designing of the new facility…”

The joint governance and leadership (including but not limited to the cross-functional teamwork) was also pointed out by the participants as an enabler. As such, complementarities were effectively leveraged.

9-M-MC: “…Active engagement and support from the leadership of both institutions, including the dean and provost of the University and the executive leadership of MCME… Robust organizational structures, including the Joint Affiliation Board (JAB) and Joint Academic Council (JAC), and a jointly appointed Director of Academic Affairs. These structures provide a solid foundation for the partnership to operate and succeed…”

10-F-MC: “…Creation of joint MBRU-Mediclinic committees and boards that provide leadership, transparency, and governance framework for the public private partnership. Leadership presence and active engagement, along with continuously expressing and exhibiting support and commitment to public private partnership…”

The contextualized, phased approach by which the journey was also repetitively brought-up by the participants.

10-F-MC: “…Change in culture and adoption/evolution of medical education frameworks within MCME…”

11-M-MC: “… a newly established university and an existing private group beginning with a single hospital, expanding to all facilities in Dubai and culminating in a Master Affiliation Agreement…”

The participants perceived the whole affiliation initiative to be quite innovative, where all involved parties exhibited substantial amount of agility and “thinking outside the box”.

2-M-MB: “…It turned out to be a thumbs up for MBRU on Innovation…”

10-F-MC: “…MBRU’s affinity to innovation enabled this unique arrangement…”

There was a consensus, among the participants, that clear, consistent communication was a success factor, and where it was missing constituted opportunities for improvement.

1-M-MB: “…MCME internal messaging was very strong: they managed to get everyone on board…”

2-M-MB: “…MBRU encouraged MCME physician staff to actively engage throughout the process, where the University has an ‘open door policy’ towards them…Additional challenges include …addressing the suboptimal degree of commitment of some physician faculty…”

To the participants, it was evident that the whole approach to managing change was anchored in effective communication.

4-M-MB: “…the patient who goes to a private hospital expects to be attended to by physicians of the highest rank (i.e., consultants). MCME used signages to notify patients of the students’ presence and now students are obviously part of the teams…”

18-F-MBMC: “…Another challenge was to ensure good lines of communication. Accordingly, committees involving key players from both sides, such as: CAC, were established…”

Both parties, according to the participants, were solution oriented, proactively addressing potential challenges and risks.

10-F-MC: “…Some of the actions taken to navigate this were formalizing the process of becoming an educator with MBRU, recruitment and engagement of medical education subject matter experts, ensuring that medical educators have certified training in medical education (e.g., homegrown ACE program)…”

11-M-MC: “…The potential resistance from patients was mitigated by the robust patient consent process; the majority of patients actually embrace students’ presence… There were concerns around the sustainability of the affiliation. This never became an issue, where the journey was marked with one success after the other…continuous communication and engagement at all stages culminating into the Master Affiliation Agreement and JAB…”

The quality of how matters were unfolding, with particular attention to the curricular delivery, was continuously monitored and evaluated.

10-F-MC: “…evaluations and feedback sessions to provide transparent/ honest feedback on the quality of education received by the medical students, and constantly reviewing and adapting the curriculum and teaching models to align with international accreditation standards…”

The journey is characterized by continuous learning and development, anchored in evidence-driven decision-making.

15-M-MBMC: “…The feedback from the students was quite encouraging. We managed to instantly act upon opportunities for improvement detected by the students. The students’ performance was on a par with set standards. In terms of the patients’ point of view, we learned that the vast majority were happy to have students in the outpatient clinics during their visits, few did not mind having students around, and in very rare cases did any one patient object to having students…”

17-M-MBMC: “…MBRU was monitoring the daily feedback from students and faculty in multiple monthly joint meetings to address the progress and difficulties that were encountered at all levels…”

The entailed capacity building was identified by the participants as a prominent enabler.

6-M-MC: “…the University invested a lot of time and resources in preparing the adjunct faculty. This was initially done by hospital visits, workshops at the hospitals, orientation programs, coaching. Some physicians were quite anxious about having to teach; we worked towards addressing their readiness, supporting them in managing their anxieties. These concerted efforts bore fruits. When the first clinical rotation began, the physicians were all set, ready, prepared…”

18-F-MBMC: “…MBRU developed the ACE as online modules, as well as holding Annual Medical Education Symposium [with free Continuing Medical Education (CME) points] that all adjuncts were invited to. MBRU also supported adjunct faculty with recording online lectures for uploading on the Learning Management System…MBRU was heavily invested and tried to standardize teaching early on by delivering multiple faculty learning and development sessions held at the different hospitals and clinics. There were small group teaching sessions, requiring active engagement, held at lunchtime when the physicians were free, or sometimes early morning before the rounds and clinics started. Basic principles and concepts were covered in those sessions [e.g., conducting a case-based discussion and a mini clinical examination, and examining a case presentation] …”

## Discussion

The rapid change in health care is not a temporary shift (4), and the pace of this change, that got accelerated since the Coronavirus disease 2019 pandemic (75), constitutes a threat to AHSs, where the sophistication of governance structures and cultures could hinder adaptation (18). Moreover, the academic culture that is at best student-centric and involves a high degree of independence needs to shift to a patient-centric model. This all calls for a culture of collaboration (17) with tighter alignment between AHSs' clinical, academic, and research missions based on an interprofessional model of care and education (80) designed to achieve better outcomes at lower cost (9). The current study sheds light on the latent potential in public private partnerships, within the context of AHSs, and on a means of leveraging action research (81) to go about forming such an affiliation. It introduced a novel conceptual framework, namely: “Public Private Affiliation Journey”, that can be deployed, within the context of AHSs, to increase the chances of success of public private partnerships and to maximize the value attained from them.

This novel conceptual framework (i.e., “Public Private Affiliation Journey”) is intended to enable foresight and reduce uncertainty, in terms of how such an affiliation journey can unfold. The current study relied on reflections made with the benefit of hindsight, describing what had taken place. The lessons learned from the entailed firsthand experiences can become a roadmap for such affiliations, which however does not necessarily lay out the trajectory exactly as it will take place. As such, it provides more visibility, raising awareness about a potential way forward and probable influencing factors. Several elements of the “Key milestones” theme resonate with Kotter's change model which has been repeatedly deployed in times of transformation in the education and healthcare sectors (82–85). The respective model starts with creating a sense of urgency, followed by forming a guiding coalition, building strategic vision, initiating change communication, removing barriers to change, generating short-term wins, sustaining change as a continuous process, and lastly incorporating change into organizational culture (83, 86). Within multi-institutional, federated AHSs (4), public private affiliations such as that reported upon in this study, constitute an agile, cost-effective solution to health professions education. This element of AHSs tends to be resource-intensive given the requirement that students, faculty, and patients be present simultaneously, along with the need to continuously reinvent the learning and teaching modes of delivery to meet the broad everchanging health needs of individuals and populations in the future (16). Knowledge transfer, on its own, is insufficient to educate and train health professionals, who are supposed to attain the knowledge and technical skills that will enable them to practice independently.

This study suggests that, in such affiliations, intentionally striving to attain each of the sequential milestones of the conceptual framework can expedite the unfolding of events. According to the introduced framework, this becomes especially true when the environment is conducive and the suggested driving forces (namely: “Aspiring for success”, “Leveraging human qualities”, and “Doing things the right way”) are propelling the movement forward. Along those lines, the literature around AHSs highlights various success factors for such affiliations (4). These include acknowledging the value that each party brings to the AHS; optimizing the environment to achieve best collective performance; having a robust university-healthcare provider affiliation agreement which effectively lays out the operational framework; developing an effective shared governance; measuring performance strategically (identifying where alignment of goals and implementation will maximize benefits for all); and establishing effective communication about achievements to facilitate recognition and new opportunities for collaboration. From a practical perspective, the firsthand experience reported upon in the current study showed that of all the things that were *done the right way*, specific attributes were characteristically enabling. These include committed and engaged leadership; a robust joint governance structure; regular standing meetings; clear, continuous communication; and concerted efforts directed towards equipping the physicians with the skills needed to be effective educators.

The semi-quantitative tally of the output of the analysis showed that the following two consecutive categories: “Seizing the opportunity” and “Arriving at a common ground”, were mentioned by the largest proportion of participants. This encourages considering those two categories as exceptionally critical milestones where decisive change in the situation occurred. These two milestones highlight a turning point in the trajectory. Prior to that, the journey took the form of an incubation phase, where a lot was lurking beneath the surface. As portrayed in the action research that has been taking place as part of the public private affiliation in a federated AHS that is reported upon in the current study, the affiliation agreement can be used to develop a framework for shared oversight of joint academic activities that enable faculty members and institutional learners to experience a harmonized environment. It has been previously suggested that within a federated AHS, three types of policies and procedures must be recognized (4): activities subject to the authority of the university, activities subject to the authority of the healthcare providing entity, and activities that are harmonized and agreed upon to be used both by the university and the healthcare providing entity. This allows for there to be clarity, among the stakeholders (including but not necessarily limited to faculty members, students/trainees, and administrators), about who has authority and jurisdiction over any matter arising.

Among the prominent attributes of the journey, that were reflected upon by this study participants, was that “necessity is the mother of invention”, as delineated in the “Observing a triggering need” category. This links well with a finding from a study which explored employees' perception about change and agility in the same context of the current study, where three themes emerged from the inductive, qualitative analysis: trigger, execution, and results (87). Moreover, “Finding a good match” category showed that the pairing institutions need to connect and be compatible on a values' level for harmony to be attained, and for the relationship between the two institutions to be sustainable over time and in the face of adversity. This interinstitutional harmony proved to enable the trust building process. According to the study's participants, the trust between MBRU and MCME, and the consequential *affiliation resilience*, is what enabled the partnership to withstand the unprecedented test of the pandemic. Similar to what was portrayed in the affiliation journey reported upon in the current study, it was previously suggested that such partnerships require first and foremost shattering of barriers and aligning of incentives (14). The value of cobranding is clear when it comes to federated AHSs (4), and the firsthand experiences reported upon in this study showed that the value of collaboration and joint investment needs to be established for the cobranding to be effective. By deliberately cultivating a culture of mindfulness, prioritising trust, collaboration, and solidarity, the affiliation was anchored in a strong foundation for organisational resilience. This finding is consistent with research indicating that organisations with cohesive teams, and engaged and empowered employees are better equipped to adapt to change (88–90).

Two of the categories within the “Key Milestones” theme, namely: “Looking ahead” and “Venturing for the right reasons”, emphasize the importance of beginning with the end in mind. There was a clear “why” to this affiliation; the desired destination was clearly defined up-front. Both entities wanted to give back to the community-at-large through contributing to medical education by creating a pipeline for future physicians, and their ultimate goal was to improve the outcomes of care. It has been suggested, by the United Kingdom's Advanced Institute for Management, that AHSs create the environment for bidirectional knowledge flow between the realms of “clinical care and research”, “education and research”, and “education and clinical care” (17). The Institute's research identifies the linkages among patient care, medical education, and research to be crucial for the successful creation of value, where this value is more than what is possibly achieved when these three elements are operating alone. This is in alignment what was portrayed as part of the “Looking ahead” milestone of the current study's conceptual framework. Hence, management of AHSs needs to focus on assuring these flows are optimal, and this involves systematically addressing the environmental barriers to collaboration (4).

One of the three categories within the “Driving forces” theme, namely: “Leveraging human qualities”, directly links to the resource-based view of health care (91, 92), which highlights the importance of harnessing strategic resources (valuable, rare, and inimitable) to enable a sustained competitive advantage. It also resonates with the common belief that people (in any one organization) are the most valuable asset. It was evident in the KOLs' feedback, in the current study, how much they needed to trust and rely on their physicians. Relevantly, it was previously suggested that it is worth reimagining the role and identity of an academic hospitalist, emphasizing customized career pathways and diverse educational roles (6).

The study also sheds light on the latent potential of deploying participatory action research (37) in the formation of public private affiliations. As previously proven, this modality, in the context of the current study, enabled bridging the gap between theory and practice by anchoring decisions in real-world experiences (81, 93). It empowered stakeholders to make evidence-based decisions through continuously collecting and analyzing data, leading to more effective strategies and improved outcomes.

This study has a few limitations. While focusing on a single affiliation (between a public medical school and a private healthcare provider) enabled the development of in-depth insights, the generalizability of the findings is limited. Their transferability is possible and encouraged; this, however, needs to be done with careful consideration of contextual variables. The results of this study encourage replicating the affiliation reported upon in other contexts, if/ when the need arises. It would be valuable to tap into the perception of KOLs of such future affiliations, with the objective of systematically identifying the similarities and differences across contexts. In addition, the qualitative narrative data, combined with the phenomenological participant-focused approach in the current study, allowed the researchers to tap into the KOLs' lived experiences which holds significant value, in terms of the research findings. However, in terms of reliability of the methodology, it would be worthwhile for future studies to deploy a mixed methods approach to research that systematically integrates qualitative with quantitative findings, ideally capturing the perceptions of more than one group of key stakeholders (e.g., students and patients). Moreover, this study enabled the development of an impression of the efficacy of the affiliation journey (through exploring perceptions of a group of key stakeholders) but not really its effectiveness, in terms of the enabled clinical learning experiences and otherwise. There is also the recall bias that might have affected the validity of the research findings, given that the KOLs were reflecting on what had taken place during the initial phase of an ongoing affiliation with the benefit of hindsight. It would be interesting for future studies to measure the extent to which the affiliation's preset objectives were attained.

## Conclusion

This study showed that there is a latent potential in forming public private partnerships, that can actually enable the formation and development of AHSs. It also showcased how the guidelines of action research can be set as the basis of the process of partnership formation, and how following those guidelines in such an endeavor maximizes value for all. In addition, it clearly brought forth the importance of having a robust governance structure with committed and engaged leadership, and clear communication channels, and of equipping the physicians with the skills needed to be effective educators. Lastly, the current study introduced the “Public Private Affiliation Journey” conceptual framework, which can be deployed in “federated” AHSs worldwide to increase the chances of success of public private partnerships and to maximize the value attained through them.

## Data Availability

The original contributions presented in the study are included in the article/Supplementary Material, further inquiries can be directed to the corresponding author.

## Appendix I: survey/semi-structured interview protocol

**Part A: Trigger**

1. Describe the (initial) **trigger**/need (internal/ external).

**Part B: Assessment of the Situation (back then)**

2. What *motivated* MBRU stakeholders to approach MCME stakeholders?

- Describe the **opportunity/ies** that MBRU stakeholders perceived (back then) around forming an affiliation between MBRU-MCME.
- For MBRU, what were the **perceived strength(s)** of MCME? Why was MCME chosen and not another healthcare provider?

3. What *motivated* MCME stakeholders, belonging to a private for-profit hospital group, to be open to collaborate with MBRU?

- Describe the **opportunity/ies** that MCME stakeholders perceived (back then) around forming an affiliation between MBRU-MCME.
- For MCME, what were the **perceived strength(s)** of MBRU?

4. What were the perceived **weakness(es)** and anticipated **challenge(s)/ threat(s)** (back then) for MBRU? How were these resolved?

5. What were the perceived **weakness(es)** and anticipated **challenge(s)**/ **threat(s)** for MCME East? How were these resolved?

**Part C: Results**

If we look at it as a system with the stakeholders and the agreement as the input, how matters unfolded as the process, what would be the **output**, **outcome**, and **impact** to both entities?

6. What were the key results on MBRU?

7. What were the key results on MCME?

8. Where there any additional **unexpected results**?

**Part D: Nature of the Relationship**

9. How would you describe the **relationship** between MBRU and MCME?

- At the very beginning of the experience
- As the journey unfolded/ time passed

10. Where is **“trust”** in the bigger scheme of things?

**Part E: Further Reflections**

11. What **actual challenge(s)** were faced and how did both sides contribute to overcoming them?

12. What have we **learned** from the experience?

13. What have been the **success factors** of the relationship?

14. What made this journey a **unique** one?
